# Amyloid precursor protein modulates macrophage phenotype and diet-dependent weight gain

**DOI:** 10.1038/srep43725

**Published:** 2017-03-06

**Authors:** Kendra L. Puig, Stephen A. Brose, Xudong Zhou, Mary A. Sens, Gerald F. Combs, Michael D. Jensen, Mikhail Y. Golovko, Colin K. Combs

**Affiliations:** 1 Department of Biomedical Sciences, University of North Dakota School of Medicine and Health Sciences, Grand Forks, ND 58202, USA; 2 Department of Pathology, University of North Dakota School of Medicine and Health Sciences, Grand Forks, ND 58202, USA; 3 Grand Forks Human Nutrition Res. Center (GFHNRC), ARS-USDA, Grand Forks, ND 58201, USA; 4 Endocrine Research Unit, Mayo Clinic, Rochester, MN 55905, USA

## Abstract

It is well known that mutations in the gene coding for amyloid precursor protein are responsible for autosomal dominant forms of Alzheimer’s disease. Proteolytic processing of the protein leads to a number of metabolites including the amyloid beta peptide. Although brain amyloid precursor protein expression and amyloid beta production are associated with the pathophysiology of Alzheimer’s disease, it is clear that amyloid precursor protein is expressed in numerous cell types and tissues. Here we demonstrate that amyloid precursor protein is involved in regulating the phenotype of both adipocytes and peripheral macrophages and is required for high fat diet-dependent weight gain in mice. These data suggest that functions of this protein include modulation of the peripheral immune system and lipid metabolism. This biology may have relevance not only to the pathophysiology of Alzheimer’s disease but also diet-associated obesity.

Obesity is characterized, in part, by excessive fat accumulation throughout the body and increased levels of adipokines and inflammatory cytokines^1^,^2^. Although they may appear as disparate conditions, the risk of Alzheimer’s disease, AD, is increased by middle age obesity, type II diabetes, and cardiovascular/cerebrovascular disease^1^,^2^,^3^,^4^,^5^,^6^,^7^,^8^,^9^,^10^,^11^,^12^,^13^,^14^,^15^,^16^,^17^,^18^,^19^,^20^,^21^, suggesting that a common mechanism of pathophysiology may exist between these conditions. For example, amyloid precursor protein, APP, cleavage to generate the aggregate-prone amyloid beta, Aβ, peptide is a hallmark of the senile plaque pathology of sporadic late onset and early onset AD^22^. However, APP expression has also recently been shown to be increased in brains and adipose tissue during diet-induced obesity^23^,^24^,^25^,^26^. More importantly, adipocyte APP and circulating Aβ levels have both been shown to increase in obese individuals^27^,^28^.

Although APP is a ubiquitously expressed transmembrane protein, its function remains unclear beyond being the parent protein of Aβ peptides. It has been suggested to function as a receptor or growth factor^29^. APP has also been hypothesized to mediate cell-cell and cell-matrix interactions^30^,^31^,^32^,^33^,^34^,^35^. We and others have shown that APP is robustly expressed in peripheral monocytes/macrophages and brain microglia with increased expression or membrane localization during proinflammatory activation and with a critical role in acquiring a reactive phenotype^36^,^37^,^38^,^39^. In addition, proteolytic fragments of APP including N-terminal secreted APP fragments^40^ and Aβ peptides^41^,^42^ have well characterized abilities to stimulate these cells. Since both obesity and AD are characterized, in part, by some form of immune dysfunction^23^,^24^,^25^,^26^ we hypothesized that a common function of APP or its metabolites is to regulate the phenotype of both peripheral macrophages and brain microglia during diet-induced obesity or AD. To test this hypothesis we focused on obesity rather than AD for this study and peripheral macrophages rather than brain microglia. A high fat diet-induced model of obesity was used with C57BL/6 wild type and APP^−/−^ mice to define the requirement for APP in regulating adipose tissue hypertrophy and macrophage activation. To further assess the tissue changes, individual cell types were isolated to better define the function of APP in regulating fatty acid uptake, inflammatory secretion, and phenotypic response.

## Results

**Figure 1:**
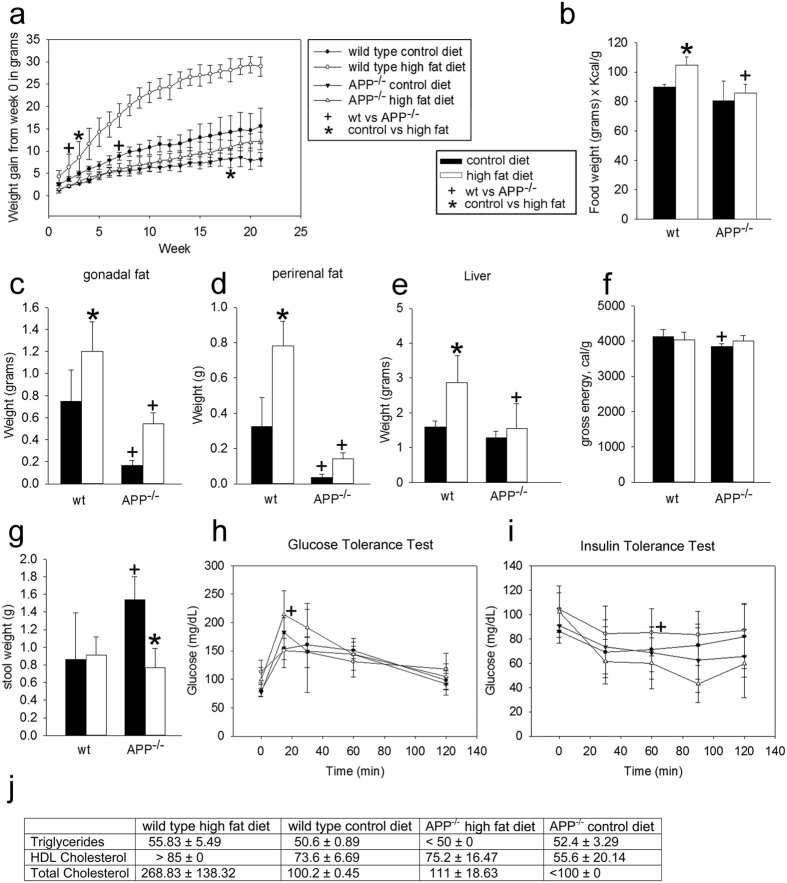
APP^−/−^ mice gained significantly less weight than wild type mice on either control or high fat diets. High fat diet feeding in wild type and APP^−/−^ mice altered weight gain (**a**), food intake × energy density (Kcal/g) (**b**), fat depot weight (**c**,**d**), liver weight (**e**), gross energy (**f**), stool weight (**g**), blood glucose and insulin tolerance levels (**h**,**i**), triglycerides, HDL, and total cholesterol (**j**). Data are expressed as mean +/− SD (n = 5 or 6).^+,^*p < 0.05.

There was a significant difference in mean starting weight between age-matched wild type and APP^−/−^ mice consistent with their smaller stature (Suppl. Fig. 1). When placed on a 21.2% by weight high fat diet, *ad libitum*, APP^−/−^ mice did not gain a significant amount of weight until after 18 weeks of high fat diet feeding unlike wild type mice which gained significantly more weight after only three weeks of high fat diet feeding when all mice were normalized to their individual starting weights (Fig. 1a). This difference is in direct correspondence to food consumed based on energy density (Kcal/g) (Fig. 1b). In fact, diminished weight gain corresponded directly with attenuated adipose tissue mass from different visceral depots and liver weights (Fig. 1c–e). Two different visceral adipose depots were measured in these studies as visceral adipose tissue alone has been shown to be responsible for the metabolic consequences of obesity^43^,^44^,^45^,^46^,^47^. Interestingly, although the APP^−/−^ mice fed a control diet consumed a similar amount of caloric content compared to wild type mice (Fig. 1b), they produced a surprisingly increased wet stool mass (Fig. 1g) of decreased caloric energy (Fig. 1f). These differences were robustly observed only in the control diet fed mice. This suggests that although the APP^−/−^ mice produced a higher mass of stool, their overall caloric energy of stool excreted was less than wild type mice consistent with their decreased caloric consumption. Glucose tolerance testing demonstrated a significantly improved sensitivity in high fat diet fed APP^−/−^ mice compared to high fat diet fed wild type mice at 15 minutes (Fig. 1h, Suppl. Fig. 2). In addition, the APP^−/−^ mice fed a high fat diet had increased insulin sensitivity overall compared to their wild type high fat diet fed counterparts (Fig. 1i, Suppl. Fig. 2). Consistent with their minimized weight gain and adipose tissue hypertrophy, APP^−/−^ mice fed a high fat diet did not demonstrate the increase in triglycerides, HDL and total cholesterol compared to their wild type controls (Fig. 1j). These data suggested an APP-mediated role in the adipose tissue hypertrophy, lipid metabolism changes, and insulin sensitivity dysfunction typically associated with high fat diet feeding.

**Figure 2:**
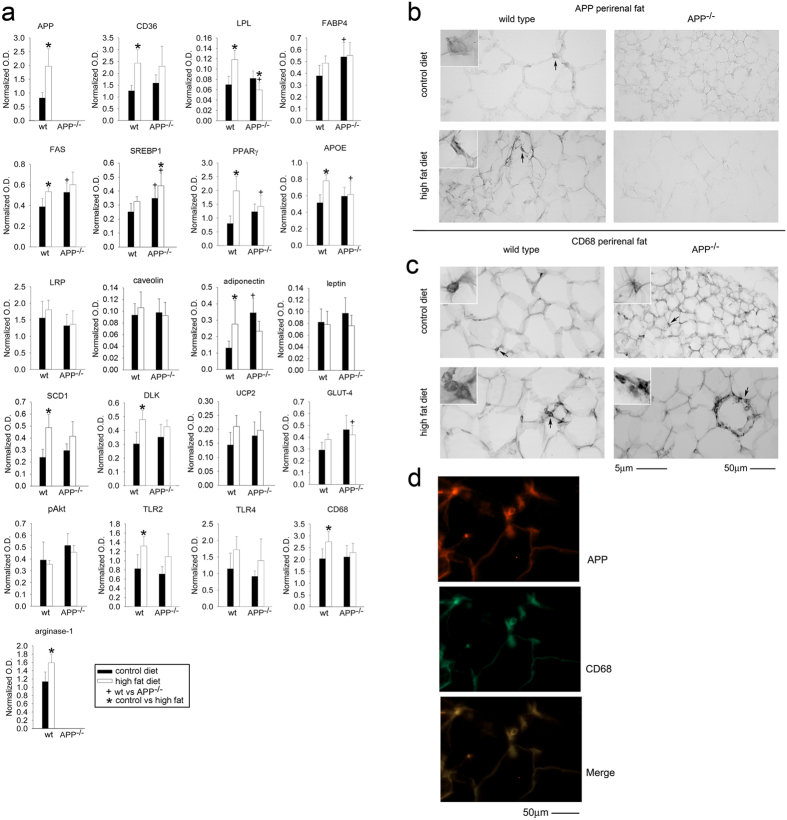
Quantification of perirenal adipose tissue protein levels in wild type and APP^−/−^ mice fed control or high fat diets. Optical densities of western blotted perirenal adipose tissue proteins from control and high fat diet samples were normalized against their respective actin loading controls (**a**). Data are expressed as mean +/− SD (n = 5 or 6)^+,^*p < 0.05. Perirenal adipose tissue was collected, sectioned, and immunostained using anti-APP (**b**) and CD68 (**c**) antibodies and antibody binding visualized using Vector VIP as the chromogen. Tissue samples were also double immunostained using anti-CD68 antibody with FITC-conjugated secondary antibody and anti-APP antibody with Texas red-conjugated secondary antibody (**d**). Representative images from 5–6 animals per condition are shown.

In order to better examine changes in lipid metabolism and immune-related proteins across genotype and diet, a visceral depot of perirenal adipose tissue was collected for western blot and immunohistochemical analyses. High fat diet feeding increased APP protein levels, as expected, in wild type mice but also many genes associated with lipid metabolism, adipose differentiation, and fatty acid transport including CD36, lipoprotein lipase (LPL), fatty acid synthase (FAS), peroxisome proliferator activated receptor γ (PPARγ), APOE, adiponectin, SCD1, and DLK. Consistent with macrophage activation and infiltration during adipose tissue hypertrophy during high fat feeding, multiple macrophage marker proteins also increased in wild type mice including TLR2, CD68 and arginase-1 (Fig. 2a, Suppl. Fig. 3). Under control diet conditions, APP^−/−^ mice had higher protein levels of fatty acid binding protein 4 (FABP4), FAS, SREBP1, and adiponectin in perirenal adipose tissue compared to wild type mice (Fig. 2a, Suppl. Fig. 3). Under high fat diet feeding conditions, APP^−/−^ mice had significantly increased levels of SREBP1 and GLUT-4 with decreased levels of LPL, PPARγ, and APOE compared to high fat diet fed wild type mice (Fig. 2a, Suppl. Fig. 3). These data demonstrated distinct differences in lipid metabolism/transport and immune-related proteins across diet and genotype that were consistent with the diminished weight gain of the APP^−/−^ mice. In recognition of the heterogeneity between different adipose tissue depots, we also compared gonadal and abdominal subcutaneous adipose tissue across diet and genotype. Although each depot had distinct changes, the trend remained that both gonadal adipose tissue (Suppl. Fig. 4) and subcutaneous adipose tissue (Suppl. Fig. 5) were significantly different across diet and genotype from wild type mice with distinct differences in lipid metabolism and transport proteins as well as immune related proteins. Increased lipid anabolic proteins with the decreased lipid deposition phenotype in the APP^−/−^ mice suggest an additional mechanism for APP involvement in adipose tissue metabolism.

To better appreciate which cell types expressed APP in the adipose tissue depots and perhaps explain the differences in animal phenotype, immunohistochemical analyses were performed. Based upon our earlier work, we expected increased APP protein levels in wild type tissue to be localized primarily to macrophages and the changes in APP^−/−^ compared to wild type tissue to be a reflection of diminished macrophage infiltration and activation^48^. Surprisingly, APP^−/−^ mice demonstrated robust macrophage immunoreactivity even without high fat diet-feeding in both perirenal (Fig. 2c) and gonadal adipose tissue (Suppl. Fig. 6). Even more interesting was the fact that adipocytes had slight immunoreactivity for APP and APP^−/−^ adipocytes demonstrated a very obvious decrease in cell volume compared to wild type mice even in mice fed the control diet in both adipose tissue types (Fig. 2b, Suppl. Fig. 6). As demonstrated previously, robust APP immunoreactivity colocalized primarily with CD68-positive macrophages in wild type mouse tissue^49^ (Fig. 2d). Therefore, the immunohistochemistry suggested that APP may have a role in regulating not only macrophage behavior but also adipocyte phenotype.

**Figure 3:**
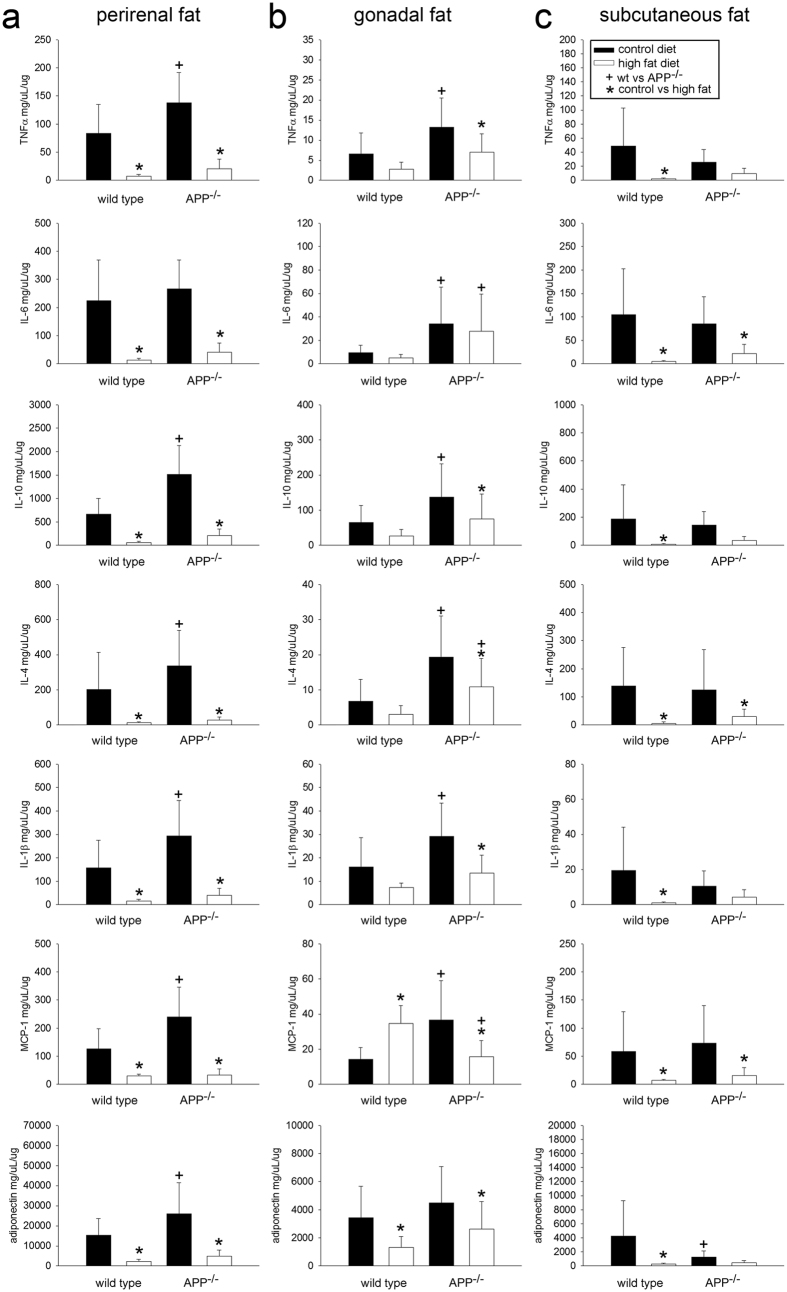
Increased inflammatory protein levels in adipose depots of APP^−/−^ mice compared to wild type mice. Perirenal (**a**), gonadal (**b**), and subcutaneous (**c**) adipose tissues were lysed to quantify IL-6, TNFα, IL-10, IL-4, IL-1β, MCP-1, and adiponectin levels via ELISA. Data are expressed as mean +/− SD (n = 5or 6),^+,^*p < 0.05.

**Figure 4:**
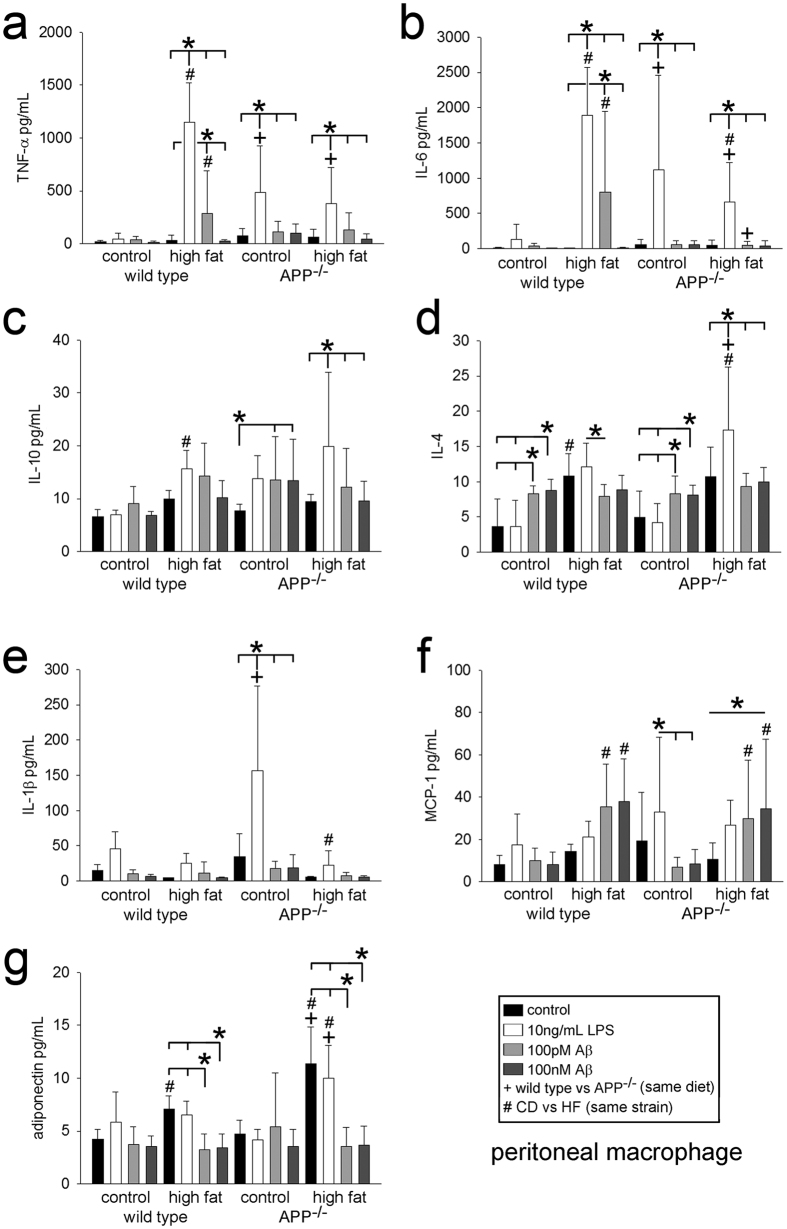
Peritoneal macrophage cytokine secretion differed between wild type and APP^−/−^ cells. Non-elicited, peritoneal macrophages from male wild type and APP^−/−^ mice fed both control or high fat diets for 22 weeks were isolated and stimulated with 10 ng/mL LPS, 100 nM Aβ 1–40, or 100 pM Aβ 1–40 overnight and the media were used to quantify TNFα (**a**), IL-6 (**b**), IL-10 (**c**), IL-4 (**d**), IL-1β (**e**), MCP-1 (**f**), and adiponectin(**g**) secretion via ELISA. Data are expressed as mean +/− SD (n = 5),^+,^*^,#^p < 0.05.

Based upon the cellular localization of APP to macrophages in wild type mice and the lack of adipose tissue hypertrophy in the APP^−/−^ mice fed the high fat diet, we expected dramatic differences in adipose tissue cytokine profiles. Indeed, comparing perirenal, gonadal, and subcutaneous adipose tissue demonstrated significant differences between genotypes and diets (Fig. 3). For example, APP^−/−^ perirenal (Fig. 3a) and gonadal (Fig. 3b) but not subcutaneous (Fig. 3c) adipose tissue demonstrated significantly higher levels of TNFα, IL-1β, IL-10, IL-4, and MCP-1 compared to wild type mice fed a control diet. This demonstrated an elevation of cytokine secretion in the APP^−/−^ mice perhaps reflective of an altered basal macrophage phenotype. In order to determine whether differences observed from adipose tissue lysates could be due to macrophage phenotype, cytokine secretions from wild type and APP^−/−^ peritoneal macrophages isolated from control and high fat diet fed mice were quantified with and without stimulation with LPS or Aβ peptide (Fig. 4). Macrophages isolated from control diet fed wild type and APP^−/−^ mice did not have dramatically different basal secretion. However, control diet fed APP^−/−^ macrophages had a significantly potentiated LPS-mediated increase in secretion of TNFα, IL-6, and IL-1β compared to wild type mice (Fig. 4a,b,e). High fat diet fed wild type mouse macrophages demonstrated an expected increase in both LPS and Aβ-stimulated TNFα and IL-6 secretion compared to their control diet fed counterparts (Fig. 4a,b). Surprisingly, high fat diet fed macrophages from APP^−/−^ mice had a significantly attenuated IL-6 secretion in response to LPS and Aβ stimulation compared to control diet fed APP^−/−^ macrophages and high fat diet fed wild type macrophages (Fig. 4b). Although peritoneal macrophages are more readily collectable from control and high fat diet fed mice, their phenotype can differ from adipose tissue macrophages. To compare whether similar phenotype differences existed across genotypes in adipose tissue macrophages, cells were grown from wild type and APP^−/−^ subcutaneous adipose tissue. Unlike the peritoneal macrophages, adipose tissue APP^−/−^ macrophages did not exhibit potentiated LPS-dependent cytokine secretion compared to wild type macrophages. Instead, they secreted reduced levels of LPS-stimulated TNFα, IL-6, and IL-4 compared to wild type cells (Suppl. Fig. 7). These data demonstrated an APP-dependent regulation of macrophage phenotypes.

Because physiological concentrations of Aβ were sufficient to stimulate macrophages in culture, we determined whether adipose tissue Aβ levels might be altered with diet as a possible consequence of the increased APP protein levels observed in wild type adipose tissue. Comparison of perirenal, gonadal, and subcutaneous adipose tissue from control and high fat diet fed mice demonstrated both Aβ 1–40 and 1–42 in all adipose tissue depots (Suppl. Fig. 8). However, the highest concentrations of either peptide were found in perirenal adipose tissue (Suppl. Fig. 8). Perhaps more importantly, high fat diet feeding slightly increased Aβ 1–40 levels in the subcutaneous adipose tissue consistent with the diet-dependent increase in APP protein levels in this depot (Suppl. Fig. 8).

**Figure 5:**
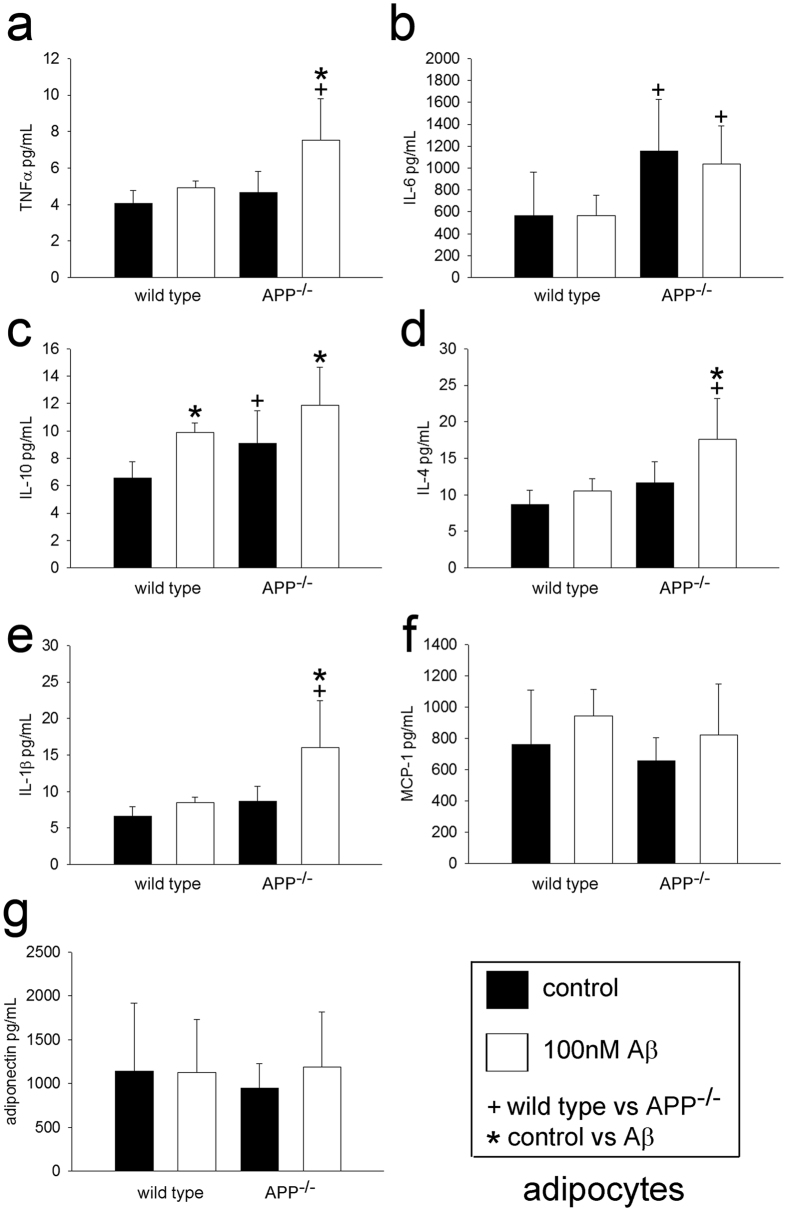
Adipocyte cytokine secretion was altered by Aβ 1–40 stimulation. Adipocytes from wild type and APP^−/−^ mice were isolated from subcutaneous adipose tissue and stimulated with or without 100 nM Aβ 1–40 overnight and media were collected to measure TNFα (**a**), IL-6 (**b**), IL-10 (**c**), IL-4 (**d**), IL-1β (**e**), MCP-1 (**f**), and adiponectin (**g**) secretion via ELISA. Data are expressed as mean +/− SD (n = 5),^+,^*p < 0.05.

Although the macrophages had clear differences between genotypes, we appreciated that adipocytes also expressed APP and the differences in weight gain and adipose tissue hypertrophy might be due to adipocyte phenotype differences as well. Wild type and APP^−/−^ adipocytes were cultured to compare protein differences. Unexpectedly, few differences in proteins involved in lipid metabolism or uptake were observed across genotypes with the exception of increased fatty acid synthase in APP^−/−^ cells (Suppl. Fig. 9). In agreement with this, there was no difference in uptake of 16:0 palmitic acid between cell types (Suppl. Fig. 9), further supporting additional mechanisms for APP regulation of lipid metabolism. However, APP^−/−^ adipocytes did basally secrete a significantly higher amount of IL-6 (Fig. 5b) and IL-10 (Fig. 5c) compared to wild type cells with no basal differences in secretion of TNFα (Fig. 5a), IL-4 (Fig. 5d), IL-1β (Fig. 5e), MCP-1 (Fig. 5f), or adiponectin (Fig. 5g) demonstrating some conservation of inflammation-related phenotype changes linked to expression of APP. Interestingly, stimulation with Aβ peptide, as a potential activating ligand in adipose tissue, resulted in increased secretion of TNFα (Fig. 5a), IL-4 (Fig. 5d), and IL-1β (Fig. 5e) preferentially in the APP^−/−^ adipocytes again demonstrating some influence of APP expression on inflammatory phenotype.

As an additional means of comparing whether the altered weight gain and adipose tissue hypertrophy were due to genotype differences in lipid uptake, storage, or release, adipocytes, adipose tissue macrophages and peritoneal macrophages were compared for their abilities to take up cholesterol or glucose, lipolysis, and lipogenesis. By these assessments, there were no differences between APP^−/−^ and wild type adipocytes (Suppl. Fig. 10). However, APP^−/−^ adipose tissue macrophages displayed increased lipogenesis compared to wild type adipose tissue macrophage (Suppl. Fig. 10). APP^−/−^ peritoneal macrophages, on the other hand, demonstrated increased ability to take up glucose compared with wild type peritoneal macrophages (Suppl. Fig. 10).

**Figure 6:**
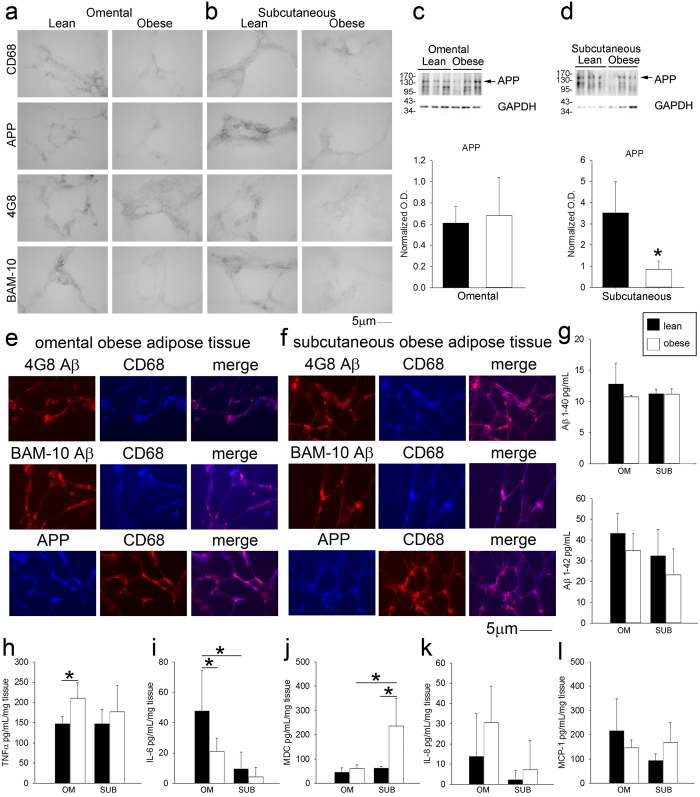
Localization and expression of APP in human lean and obese adipose tissue. Human lean and obese omental (**a**,**e**) and subcutaneous (**b**,**f**) adipose tissues were sectioned and immunostained using anti-APP, CD68, Aβ (4G8, and BAM-10) antibodies and antibody binding visualized using Vector VIP as the chromogen or Alexa fluor-conjugated secondary antibodies for double labeling. Omental (**c**) and subcutaneous (**d**) adipose tissues were also lysed, resolved by 10% SDS-PAGE and western blotted using anti-APP and GAPDH (loading control) antibodies. Arrowheads indicate bands of interest when nonspecific bands are present. Antibody binding was visualized by chemiluminescence. Optical densities of the western blotted proteins were normalized against their respective GAPDH loading controls. Lean and obese omental and subcutaneous tissue was lysed to quantify soluble Aβ 1–40 and Aβ1-42 (**g**), TNFα (**h**), IL-6 (**i**), MDC (**j**), IL-8 (**k**), and MCP-1 (**l**) levels via ELISA. ELISA values were normalized to tissue weight. Data are expressed as mean +/− SD (n = 5) *p < 0.05.

In order to determine whether the diet-dependent increase in macrophage APP and adipose tissue Aβ in the murine high fat diet feeding paradigm was consistent with the human condition, we compared visceral omental and subcutaneous adipose tissue from lean and obese human subjects. Human tissue demonstrated both APP and Aβ immunoreactivity in both omental (Fig. 6a,e) and subcutaneous (Fig. 6b,f) tissue. Consistent with the murine data, APP immunoreactivity co-localized with the macrophage marker, CD68, in both omental (Fig. 6e) and subcutaneous (Fig. 6f) tissue. Cytokine levels were quantified from both lean and obese adipose tissue to simply define differences in inflammatory profile. Obese adipose tissue demonstrated some expected changes such as elevated TNFα levels in omental but not subcutaneous adipose tissue (Fig. 6h) with an unexpected decrease in IL-6 levels in obese omental tissue (Fig. 6i). Protein levels of full length APP were not different in lean compared to obese omental tissue (Fig. 6c) although subcutaneous adipose tissue APP levels were significantly lower in obese compared to lean samples (Fig. 6d). Because this decrease may suggest increased processing of APP to proteolytic fragments, levels of Aβ in each tissue were quantified. Surprisingly, in spite of the attenuated subcutaneous adipose tissue APP, there were no significant increases in Aβ 1–40 or 1–42 peptide levels between lean and obese omental and subcutaneous human adipose tissue. This is perhaps a reflection of small sample size, decreased production, or alternative processing in human tissue (Fig. 6g).

## Discussion

Our findings demonstrate that APP expression is critical for the weight gain and adipose tissue hypertrophy that occurs in a murine model of high fat diet-induced obesity (Fig. 1a; Fig. 2b,c). We observed a high fat diet-induced increase in APP protein levels in two visceral adipose tissue depots (Fig. 2a; Suppl. Fig. 4), particularly in macrophages (Fig. 2d). This correlated with detectable levels of both Aβ 1–40/42 in all depots and a slight increase in Aβ 1–40 levels in high fat diet fed subcutaneous adipose tissue (Suppl. Fig. 8). The visceral APP^−/−^ adipose tissue depots demonstrated select elevations of cytokines in animals fed a control diet compared to their wild type counterparts (Fig. 3a,b). This correlated with altered protein levels of a plethora of proteins involved in lipid metabolism and immune cell phenotype (Fig. 2a, Suppl. Fig. 4). Similarly, APP^−/−^ peritoneal macrophages from control diet fed mice demonstrated increased LPS-stimulated secretion of several cytokines compared to control diet fed wild type macrophages (Fig. 4a,b,e). In fact, even APP^−/−^ adipocytes had elevated secretion of some cytokines compared to wild type adipocytes (Fig. 5b,c). On the other hand, APP^−/−^ and wild type adipocytes were not significantly different in their abilities to take up cholesterol, release fatty acid, or stimulate lipid synthesis (Suppl. Figs 9,10). Human tissue analysis demonstrated adipose tissue APP expression and colocalization to macrophages (Fig. 6a,e,f) and detectable Aβ (Fig. 6g) similar to the mouse tissue findings. However, perhaps due to species differences or limitation in sample size obese omental visceral adipose tissue did not demonstrate an increase in APP protein levels similar to gonadal or perirenal adipose tissue in high fat diet fed mice.

This more robust change in immune cell phenotype is consistent with our prior work demonstrating a role for APP in regulating the behavior of monocytes, microglia, and macrophage^38^,^39^,^50^. Others have reached a similar conclusion particularly with regard to microglia^51^. However, the role of APP in regulating any particular adipose tissue inflammatory phenotype was quite complex with differences between the adipose tissue depots. In fact, in spite of a consistent difference in secretion profile when comparing wild type to APP^−/−^ macrophages there was not a uniform secretory phenotype between APP^−/−^ macrophages. This is not unexpected given their functional differences as peritoneal versus adipose tissue macrophages. Perhaps most interesting were the differences observed in the peritoneal macrophages since these cells were acutely isolated from control and high fat diet fed animals without the extended culture period required for both adipose tissue adipocytes and macrophages. The APP^−/−^ peritoneal macrophages demonstrated a robust potentiation of LPS-stimulated cytokine secretion compared to wild type cells that was consistent with basally elevated adipose tissue cytokines in the mice. Interestingly, even adipocytes from APP^−/−^ mice demonstrated select elevations in cytokine sections. This suggests an intriguing role for APP or a proteolytic fragment in basally down-regulating the immune system, at least in the periphery. Consistent with this idea, we have observed in the intestines of APP^−/−^ mice compared to wild types, reduced macrophage numbers and altered cytokine profiles^52^. Similar to our findings, others have demonstrated that Aβ is a potent anti-inflammatory molecule in select paradigms. Intraperitoneal injections of Aβ reduce inflammation and symptoms in the experimental autoimmune encephalomyelitis mouse model of multiple sclerosis with exacerbated disease in APP^−/−^ mice^53^. Collectively, it appears that APP and perhaps its proteolytic fragments have a critical role in regulating macrophage phenotype. At this point, however, it is not clear whether the immune changes observed are directly related to the attenuated weight gain and adipose tissue hypertrophy observed in the high fat diet fed APP^−/−^ mice. Macrophage selective assessment of APP will help resolve whether its function in these cells is responsible for the adipose tissue hypertrophy that occurs during high fat diet feeding.

It is not clear what cell type was responsible for the observed increase in subcutaneous adipose tissue Aβ in wild type mice fed a high fat diet. Although we did not observe dramatic differences in adipocyte function across genotypes we cannot rule out the possibility that APP or a fragment such as Aβ may have a role in directly regulating lipid homeostasis and transport. For example, our prior work in the human Caco-2 enterocyte line demonstrated an ability for Aβ or sAPP peptide stimulation to decrease and increase cholesterol uptake, respectively, in these cells^54^. Others have also shown a relationship between elevated peripheral Aβ and altered lipid homeostasis. The TgCRND8 line increases plasma Aβ levels in parallel with very low density lipoprotein triglyceride levels before plaque deposition^55^. The human HepG2 cell line secretes Aβ as a lipoprotein complex supporting a possible role for Aβ in lipid transport^56^. In addition, high fat diet increases intestinal epithelial Aβ immunoreactivity and secretion of Aβ-containing chylomicrons in C57BL/6 mice^57^,^58^,^59^,^60^. Finally, intravenously injected Aβ 1–40 can associate with chylomicron-like particles for transport to the brain^61^. As already mentioned, adipocyte APP has been shown to increase in obese humans correlating with circulating Aβ 1–40 levels^28^,^62^. More importantly, adipocytes have been shown to directly secrete Aβ^63^. Future work with APP fragment stimulation *in vivo* or cell-specific APP deletion will better resolve the questions of whether an adipocyte specific effect of APP or its fragments are needed for adipose tissue hypertrophy during high fat diet feeding.

A major deficiency in the mechanistic understanding of how mutations in APP contribute to AD has been the fact that the function of APP has been poorly resolved. Although convincing evidence supports the fact that Aβ peptide contributes to the pathophysiology of AD^22^, our data suggests that the biology of the precursor APP should also be considered. At the very least, unraveling the cell-specific function of APP in parallel with any proteolytic processing by adipocytes and macrophages during high fat diet feeding may offer insight into events that occur during obesity that may be extrapolated to AD. This data demonstrates a specific function of APP or its metabolites is involved in the changes that occur during high fat diet-induced obesity.

## Methods Summary

Under approved protocols, six weeks of age, 11 male C57BL6/J wild type mice and 10 male APP^−/−^ mice^48^ were placed on either a 21.2% by weight high fat diet or a 5.5% by weight regular fat diet, *ad libitum*^49^. After 22 weeks of diet feeding visceral (perirenal and gonadal) and abdominal subcutaneous adipose tissues were collected, weighed, and prepared for analyses. Western blotting, immunohistochemistry, ELISA, and stool weight analyses were performed as previously described^48^,^49^,^64^. Macrophages and adipocytes were isolated as previously described^48^,^49^,^65^. Fatty acid uptake was performed from cell cultures as previously described^38^,^48^,^49^,^66^. Gross energy measurements were performed by bomb calorimetry. Data were analyzed using an unpaired t-test with Welch correction when comparing only wild type to APP^−/−^, two-way repeated measures ANOVA with Holm-Sidak post hoc test for weight gain and glucose measurements, or a one-way ANOVA with Holm-Sidak post hoc test for stimulated wild type cells or two-way ANOVA with Holm-Sidak post hoc test when comparing diets or stimulations between wild type and APP^−/−^ animals/cells.

## Online Methods

### Materials and Methods

#### Materials

Anti-mouse IgM (goat), anti-rabbit (goat), anti-goat (bovine), anti-rat (goat), and anti-mouse (bovine) horseradish peroxidase-conjugated secondary antibodies were purchased from Santa Cruz Biotechnology (Santa Cruz, CA, USA). FABP4, CD36, PPARγ, SREBP1, UCP2, FXR, LXRα, LXRβ, TLR4, caveolin, arginase-1, VLDLR, GAPDH, α-tubulin and actin antibodies were purchased from Santa Cruz Biotechnology (Santa Cruz, CA, USA). Elite Vectastain ABC Avidin and Biotin, Vector VIP, Vector DAB, biotinylated anti-rabbit, anti-mouse, and anti-rat antibodies were purchased from Vector Laboratories Inc (Burlingame, CA, USA). CD68 antibody was purchased from AbD Serotec (Oxford, UK). pAKT, and AKT antibodies were purchased from Cell Signaling Technology Inc (Danvers, MA, USA). APP (Y188), TNFα, FAS, LPL, DLK, FABP4, Adiponectin, Leptin, and APOE were purchased from Abcam Inc (Cambridge, MA, USA). TLR2 antibody was purchased from Imgenex (San Diego, CA, USA). Aβ clone 4G8 was purchased from Covance (Emeryville, CA, USA). BAM-10 was purchased from Sigma-Aldrich Corp. (St. Louis, MO, USA). GLUT-4 antibody was purchased from EMD Millipore (Billerica, MA, USA). SCD1 antibody was purchased from Thermo Fisher Scientific Inc. (Rockford, IL, USA). Alexa Fluor 594 donkey anti-mouse IgG, Alexa Fluor 594 donkey anti-rat IgG, Alexa Fluor 350 goat anti-rat IgG, and Alexa Fluor 350 donkey anti-rabbit IgG antibodies were purchased from Life Technologies (Grand Island, NY, USA). LRP (H4 clone) antibody was generously provided by Dr. Isa Hussaini (U. Virginia, Charlottesville, USA).

#### Mice

APP^tm1Dbo^/J homozygous (APP^−/−^) mice and wild type (C57BL6) mice were purchased from Jackson Laboratory (Bar Harbor, ME, USA). Mice were provided food and water *ad libitum* and housed in a 12 hour light/dark cycle. The investigation conforms to the *Guide for the Care and Use of Laboratory Animals* published by the US National Institutes of Health (NIH Publication No. 85-23, revised 1996). Animal use was approved by the University of North Dakota IACUC.

#### High Fat vs. Control Diet Feeding

At six weeks of age, 11 male C57BL6 wild type mice and 10 male APP^−/−^ mice were placed on either a 21.2% by weight high fat diet (Harlan Teklad TD.88137) or a 5.5% by weight regular fat diet (Harland Teklad 8640), *ad libitum*. Animals were weighed each week for 22 weeks. Food was weighed each week during weeks 13–17. Food consumed was multiplied by the energy density (Kcal/g) 3.0 Kcal/g for control diet and 4.5 Kcal/g for high fat diet. After 22 weeks of diet feeding the animals were perfused with PBS and liver, visceral (perirenal and gonadal) fat, and subcutaneous fat was collected.

#### Glucose, Triglycerides, HDL and Total Cholesterol Measurements

At 22 weeks mice were fasted for 6 hrs (water only) and then glucose, triglycerides, HDL and total cholesterol were measured using a CardioChek meter (Test Medical Symptoms @ Home, Inc, Maria Stein, OH, USA) and corresponding strips via tail lancet or True Result glucose meter and corresponding test strips. After baseline measurements were recorded mice were orally gavaged with 58 mg/mouse of glucose followed by 15, 30, 60, 120 min glucose measurements. One week later, mice were again fasted for 5–6 hrs (water only) and then glucose was measured. After baseline measurements were recorded mice were injected intraperitoneally with 0.02157 U/mouse insulin followed by 15, 30, 60, 120 min glucose measurements.

#### Stool Weight

Prior to collection, total stool weight was measured by placing the animals in a clean empty cage and collecting all stool produced over a 1 hr time period at the same time of day per animal.

#### Bomb Calorimetry

Fecal samples were air-dried and mixed with a mortar and pestle to uniform particle size. Total energy was determined in 0.5 g samples by adiabatic oxygen bomb calorimetry using a Parr 6200 Isoparibol Calorimeter (Parr Instrument Company, Moline, Ill.)

#### Human Adipose Tissue

Human adipose tissue was obtained from Dr. Michael D. Jensen at the Mayo Clinic Rochester, MN. Ten male samples were obtained, five lean (BMI 23.2–26.91) and five obese (BMI 40.25–51.90) from both a subcutaneous and omental fat depot were examined. Written, informed consent was obtained from all participants. The study was approved by the Institutional Review Board of the Mayo Clinic. These studies were done in compliance with the principles of the Declaration of Helsinki.

#### Peritoneal Macrophage Isolation and Stimulation

Macrophages were isolated by washing the peritoneal cavity with sterile PBS. The PBS was centrifuged, cells were counted and plated 0.5 × 10^6^ cells/well onto tissue culture plastic for 3 hours in DMEM/F12. Plates were rinsed with ice cold DMEM/F12, any floating debris was removed.

#### Adipocyte and Adipocyte Macrophage Isolation and Stimulation

Subcutaneous abdominal adipose tissue was removed from the mice, rinsed with sterile Hanks basal saline solution (HBSS), cut into small pieces, placed in 35 mm plate, incubated for 1 hr @ 37 C on a rocker in sterile enzyme solution (5 mM glucose, 1.5% BSA, 5 mg collagenase in HBSS). Following incubation adipocytes were sucked up, placed in Eppendorf tubes, spun at 186 × g for 2 min, the upper layer was removed for adipocyte and fibroblast isolation and the lower layer was removed for macrophage isolation, both layers were resuspended in sterile HBSS to rinse, spun at 186 × g for 2 min, again separating the lower and upper layers. The upper layer containing adipocytes and fibroblasts was resuspended in DMEM/F12 + 10% FBS + 5% horse serum + antibiotics (PSN) and added to a T-25 flask with media or plated in 96 well plates, covered with sterile parafilm. The cells were grown in inverse position (bottom tissue culture treated side up) cell culture for 2 weeks. In T-75 flasks and 96 well plates, adipocytes in suspension floated to the tissue culture treated side up allowing the cells to adhere while fibroblasts sunk to the bottom non tissue culture treated side. For adipocyte use, the cultures were returned to right side up. The lower layer from the spin that contained adipose tissue macrophages was resuspended in DMEM/F12 without serum for 3 hours. Plates were then rinsed with ice cold DMEM/F12 and any floating debris was removed.

#### Western Blotting

Tissues or cells were lysed using ice cold RIPA buffer (20 mM Tris, pH 7.4, 150 mM NaCl, 1 mM Na_3_VO_4_, 10 mM NaF, 1 mM EDTA, 1 mM EGTA, 0.2 mM phenylmethylsulfonyl fluoride, 1% Triton, 0.1% SDS, and 0.5% deoxycholate) with protease inhibitors (AEBSF 104 mM, Aprotinin 0.08 mM, Leupeptin 2.1 mM, Bestatin 3.6 mM, Pepstatin A 1.5 mM, E-64 mM). To remove insoluble material, cell lysates were sonicated and centrifuged (14,000 rpm, 4 °C, 10 min). The Bradford method (Bradford, 1976) was used to quantitify protein concentrations. Proteins were resolved by 7–10% SDS-PAGE and transferred to polyvinylidene difluoride membranes for Western blotting using anti- CD36, PPARγ, SREBP1, SREBP2, UCP2, FXR, LXRα, LXRβ, TLR4, CRP, caveolin, arginase-1, VLDLR, CD68, GAPDH, pAKT, AKT, APP (Y188), TNFα, GLUT-4, FAS, LPL, DLK, FABP4, adiponectin, leptin, APOE, TLR2, SCD1, LRP, α-tubulin and actin antibodies. Antibody binding was detected with enhanced chemiluminescence (GE Healthcare, Piscataway, NJ). In some instances, blots were stripped in 0.2 NaOH, 10 min, 25 °C, for reprobing. Western blots were quantified using Adobe Photoshop software. Optical density of bands were normalized against their respective loading controls and averaged (+/−SD).

#### Immunohistochemistry

Flash frozen adipose tissue samples were serially cryosectioned (20 μm) for immunostaining with anti-APP, CD68, 4G8, or BAM-10 antibodies or respective secondary only antibodies. Antibody binding in fat and liver was visualized using Vector VIP as chromogens (Vector Laboratories, Burlingame, CA). Images were taken using an upright Leica DM1000 microscope and Leica DF320 digital camera system. Figures were made using Adobe Photoshop software.

#### Immunofluorescence

Sectioned tissue was immunostained using anti-APP, CD68, 4G8 or BAM-10 antibodies or respective secondary only antibodies. Antibody binding was visualized using Alexa Fluor 594 or 350 secondary antibodies. Images were taken using a Zeiss LSM 510 Meta Confocal Microscope.

#### Tissue Enzyme-linked Immunosorbent Assay (ELISA)

Tissue was weighed then lysed in ice cold RIPA buffer with protease inhibitors. To remove insoluble material tissue lysates were sonicated and centrifuged. Cells were stimulated overnight in DMEM/F12 with or without 10 ng/mL LPS, 100 pM or 100 nM Aβ 1–40. Media from stimulated cells or tissue lysates were then plated for quantifying TNF-α, IL-6, IL-10, IL-4, IL-1β, MDC, IL-8, MCP-1, and adiponectin according to the manufacturer protocol (R&D Systems, Minneapolis, MN). Tissue lysates were also used for commercial Aβ 1–40 and Aβ 1–42 ELISA (Life Technologies, Grand Island, NY, USA).

#### Uptake/lipid composition for cell cultures

[1–^14^C]16:0 were dissolved in ethanol and had a final specific activity of 55 nCi/nmol. Cells were incubated in serum free media containing fatty acid tracer (50 nCi/ml) for 5, 15 and 30 min at 37 °C. Ethanol concentration in the medium was 0.2%. At the end of incubation, cells were washed twice with ice-cold phosphate-buffered saline and extracted with hexane:2-propanol (3:2) and an aliquot of lipid extract was assayed for radioactivity using liquid scintillation counter.

#### Cholesterol Uptake, Glucose Uptake, Glycerol Release, and Adipogenesis

Plated cells were used following the manufacturer protocol for Cholesterol Uptake Cell-Based Assay Kit, Glucose Uptake Cell-Based Assay Kit, Adipolysis Assay Kit and Adipogenesis Assay Kit Cayman Chemical (Ann Arbor, MI, USA).

#### Statistical Analysis

Data were analyzed using an unpaired t-test with Welch correction when comparing only wild type to APP^−/−^, two-way repeated measures ANOVA with Holm-Sidak post hoc test for weight gain and glucose measurements, or two-way ANOVA with Holm-Sidak post hoc test when comparing diets or stimulations between wild type and APP^−/−^ animals/cells.

## Additional Information

**How to cite this article**: Puig, K. L. *et al*. Amyloid precursor protein modulates macrophage phenotype and diet-dependent weight gain. *Sci. Rep.* **7**, 43725; doi: 10.1038/srep43725 (2017).

**Publisher's note:** Springer Nature remains neutral with regard to jurisdictional claims in published maps and institutional affiliations.

## Supplementary Material

Supplementary Figures
