# Supplementary material for: Amyloid precursor protein modulates macrophage phenotype and diet-dependent weight gain
Source: Sci Rep. 2017 Mar 6;7:43725. doi: 10.1038/srep43725 (PMC5338020; doi:10.1038/srep43725)
Supplement: Supplementary Figures [file srep43725-s1.doc]

**Amyloid precursor protein modulates macrophage phenotype and diet-dependent weight gain**

Kendra L. Puig, Stephen A. Brose, ^1^Xudong Zhou, ^1^Mary A. Sens, ^2^Gerald F. Combs, Jr. ^3^Michael D. Jensen, Mikhail Y. Golovko and Colin K. Combs

Department of Biomedical Sciences, University of North Dakota School of Medicine and Health Sciences, Grand Forks, ND 58202, ^1^Department of Pathology, University of North Dakota School of Medicine and Health Sciences, Grand Forks, ND 58202, ^2^Grand Forks Human Nutrition Res. Center (GFHNRC), ARS-USDA, Grand Forks ND 58201, ^3^Endocrine Research Unit, Mayo Clinic, Rochester, MN 55905

**Supplemental Figure 1. APP^-/-^ mice weighed significantly less than wild type mice at 6 weeks old.** APP^-/-^ mice weighed less at the start of the feeding paradigm. Data are expressed as mean +/- SD (n = 5 or 6) ^+^p<0.05.

**
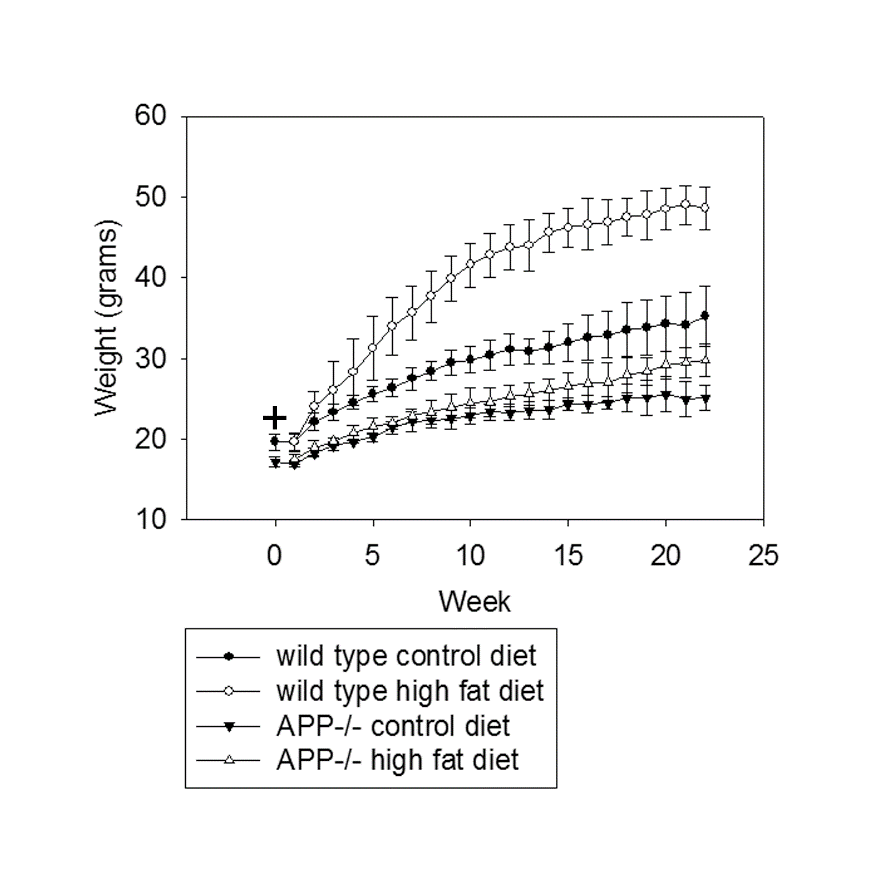
**


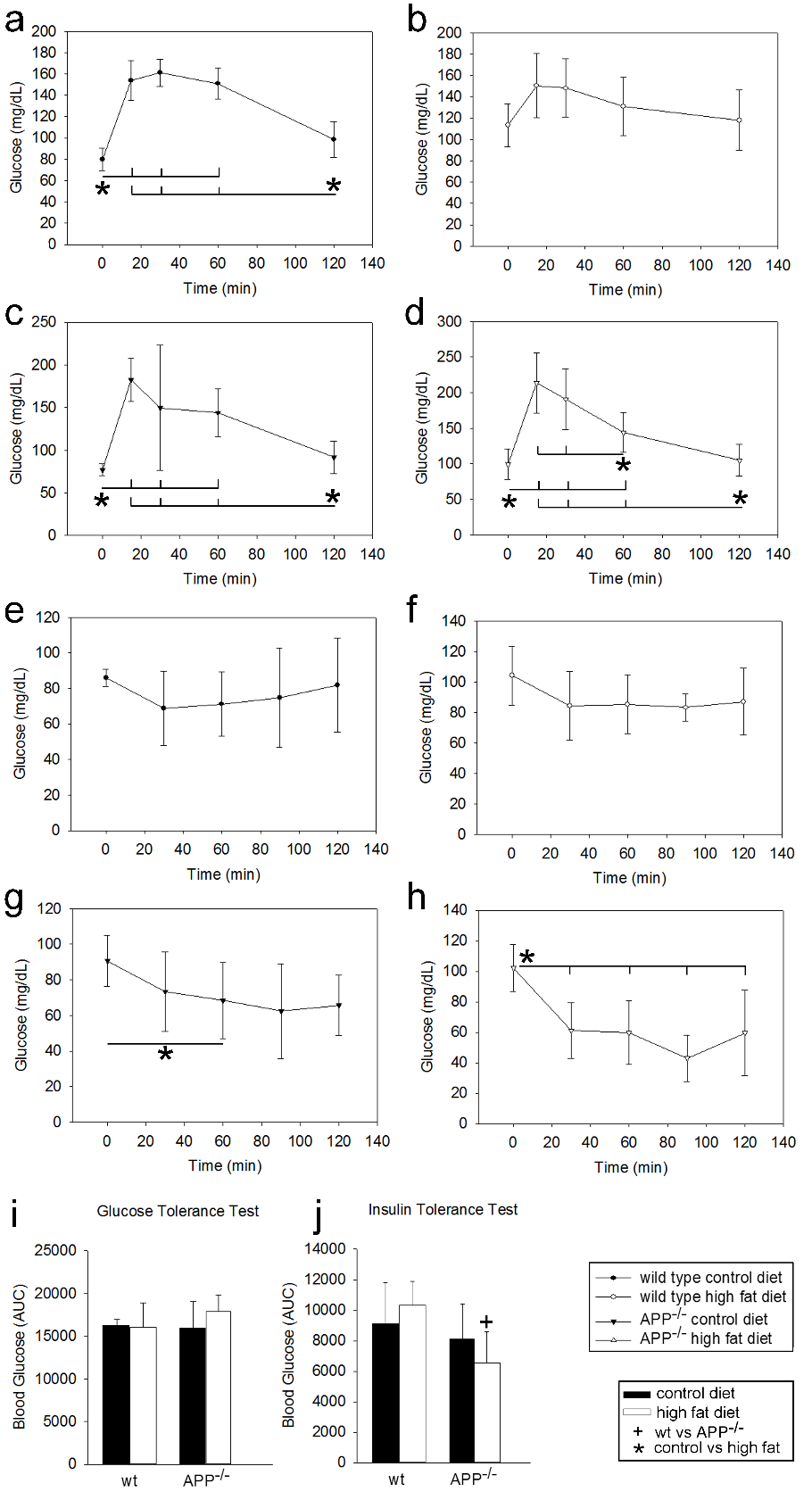
**Supplemental Figure 2. Measurement of fasting blood glucose levels in wild type and APP^-/-^ mice on control or high fat diets.** After 22 weeks of diet feeding mice were fasted for 5-6hrs (water only) and then glucose was measured. After baseline measurements were recorded mice were orally gavaged with 58mg/mouse of glucose followed by 15, 30, 60, 120 min glucose measurements. (a) Wild type mice on control diet, (b) Wild type mice on high fat diet, (c) APP^-/-^ mice on control diet, (d) APP^-/-^ mice on high fat diet. One week later, mice were again fasted for 5-6hrs (water only) and then glucose was measured. After baseline measurements were recorded mice were injected intraperitoneally with 0.02157U/mouse insulin followed by 15, 30, 60, 120 min glucose measurements. (e) Wild type mice on control diet, (f) Wild type mice on high fat diet, (g) APP^-/-^ mice on control diet, (h) APP^-/-^ mice on high fat diet. Area under the curve calculations were made based on each animals glucose curves for glucose tolerance (i) and insulin tolerance (j). Data are expressed as mean +/- SD (n = 5 or 6) ^+^,*p<0.05.

**Supplemental Figure 3.** **Western blots of perirenal adipose tissue protein levels in wild type and APP^-/-^ mice fed control or high fat diets.** Perirenal adipose tissue was lysed, resolved by 7-10% SDS-PAGE and western blotted using anti-APP, CD36, LPL, FABP4, FAS, SREBP1, PPARγ, APOE, LRP, caveolin, adiponectin, leptin, SCD1, DLK, UCP2, GLUT-4, pAkt, Akt, TLR2, TLR4, CD68, arginase-1, and actin (loading control) antibodies. Arrowheads indicate bands of interest when nonspecific bands are present. Antibody binding was visualized by chemiluminescence.

**
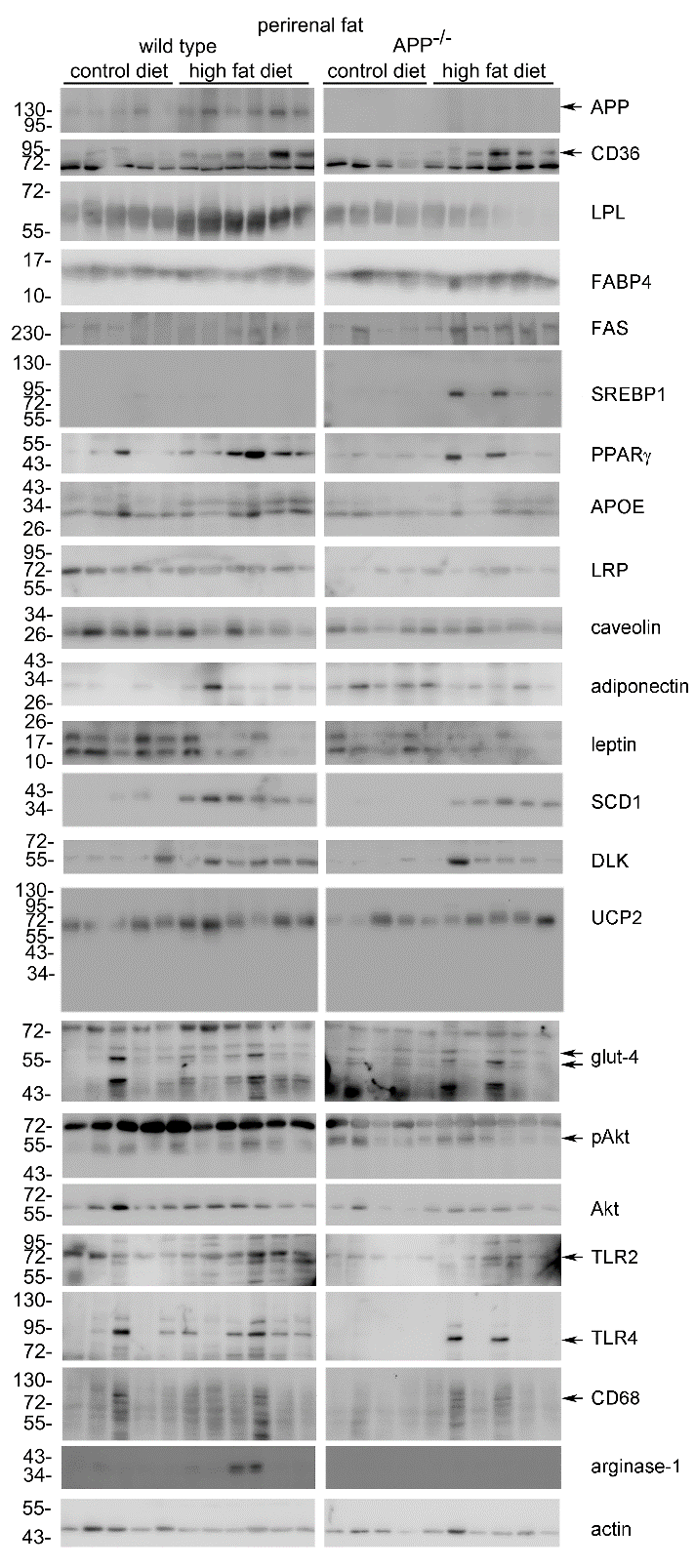
**

**Supplemental Figure 4.** **Quantification of gonadal adipose tissue protein levels in wild type and APP^-/-^ mice fed control or high fat diets.** Gonadal adipose tissue was lysed, resolved by 7-10% SDS-PAGE and western blotted using anti-APP, CD36, LPL, FABP4, FXR, LXRα, LXRβ, FAS, SREBP1, PPARγ, APOE, LRP, caveolin, adiponectin, VLDLR, SCD1, DLK, UCP2, glut-4, pAkt, Akt, TLR2, TLR4, CD68, arginase-1, and actin (loading control) antibodies. Arrowheads indicate bands of interest when nonspecific bands are present. Antibody binding was visualized by chemiluminescence. Optical densities of the western blotted gonadal adipose tissue proteins from the control and high fat diet samples were normalized against their respective actin loading controls. Data are expressed as mean +/- SD (n = 5 or 6) ^+^,*p<0.05.

**
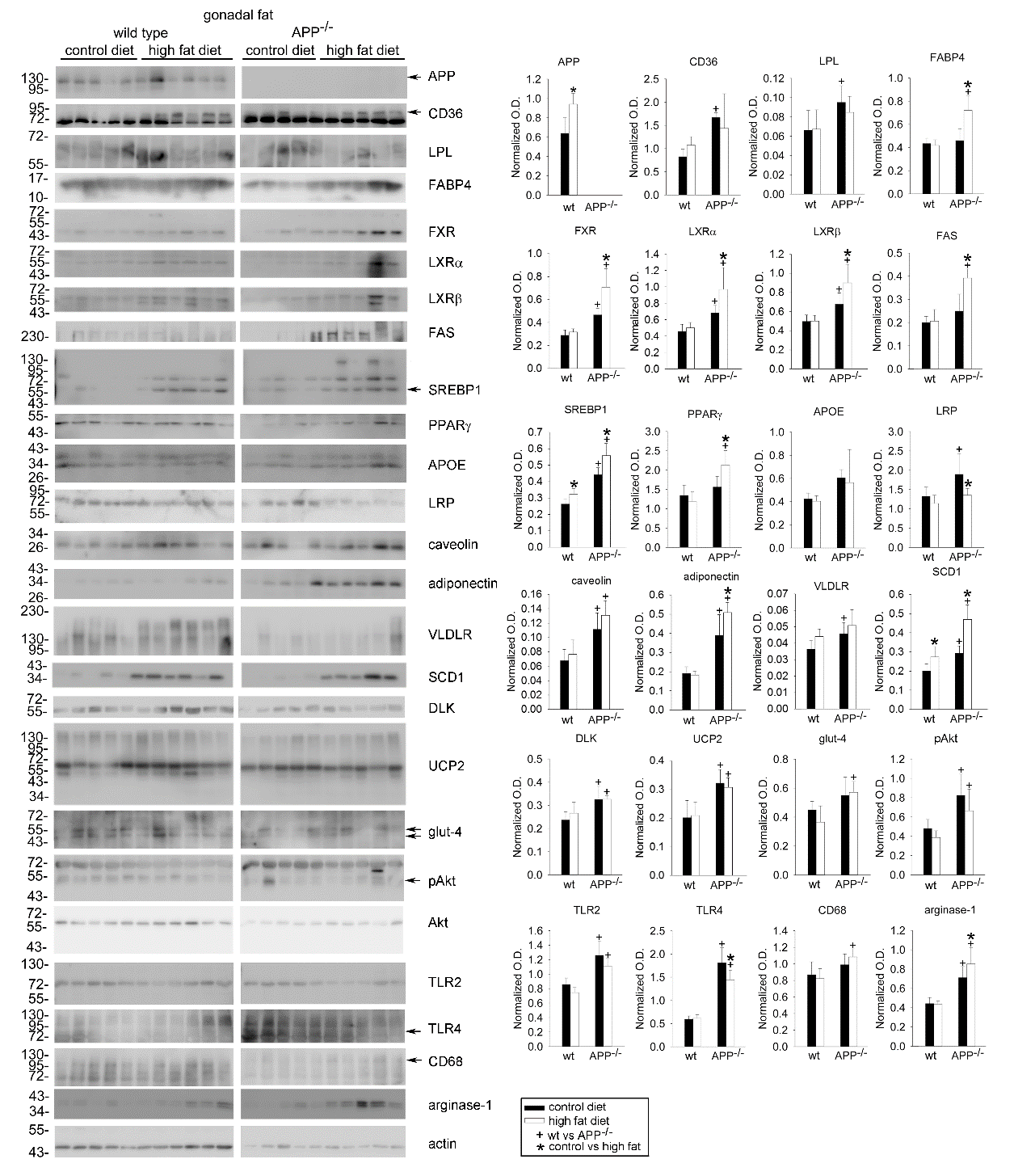
**

**Supplemental Figure 5.** **Quantification of subcutaneous adipose tissue protein levels in wild type and APP^-/-^ mice fed control or high fat diets.** Subcutaneous adipose tissue was lysed, resolved by 7-10% SDS-PAGE and western blotted using anti-APP, CD36, LPL, FABP4, FXR, LXRβ, FAS, SREBP1, PPARγ, APOE, LRP, caveolin, adiponectin, Leptin, VLDLR, SCD1, DLK, UCP2, glut-4, pAkt, Akt, TLR2, CD68, arginase-1, and actin (loading control) antibodies. Arrowheads indicate bands of interest when nonspecific bands are present. Antibody binding was visualized by chemiluminescence. Optical densities of the western blotted subcutaneous adipose tissue proteins from the control and high fat diet samples were normalized against their respective actin loading controls. Data are expressed as mean +/- SD (n = 5 or 6) ^+^,*p<0.05.

**
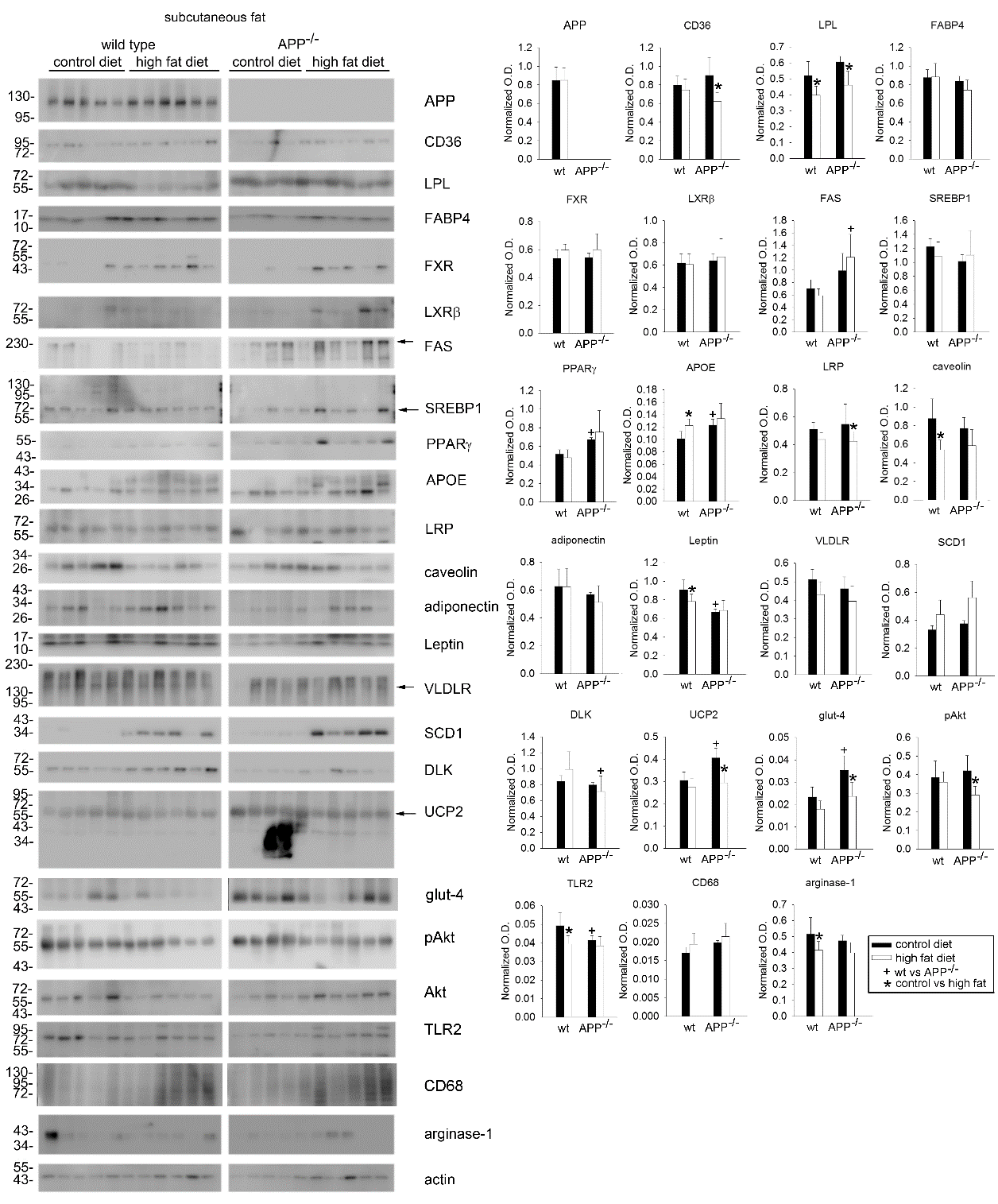
**

**Supplemental Figure 6. CD68 and APP immunoreactivity colocalized in adipose tissue and CD68 immunoreactivity increased in gonadal adipose tissue of wild type and APP^-/-^ mice fed a high fat diet.** (A) Gonadal perirenal adipose tissue was collected, sectioned, and immunostained using anti-APP and CD68 antibodies and antibody binding visualized using Vector VIP as the chromogen. Representative images from 5-6 animals per condition are shown.

**
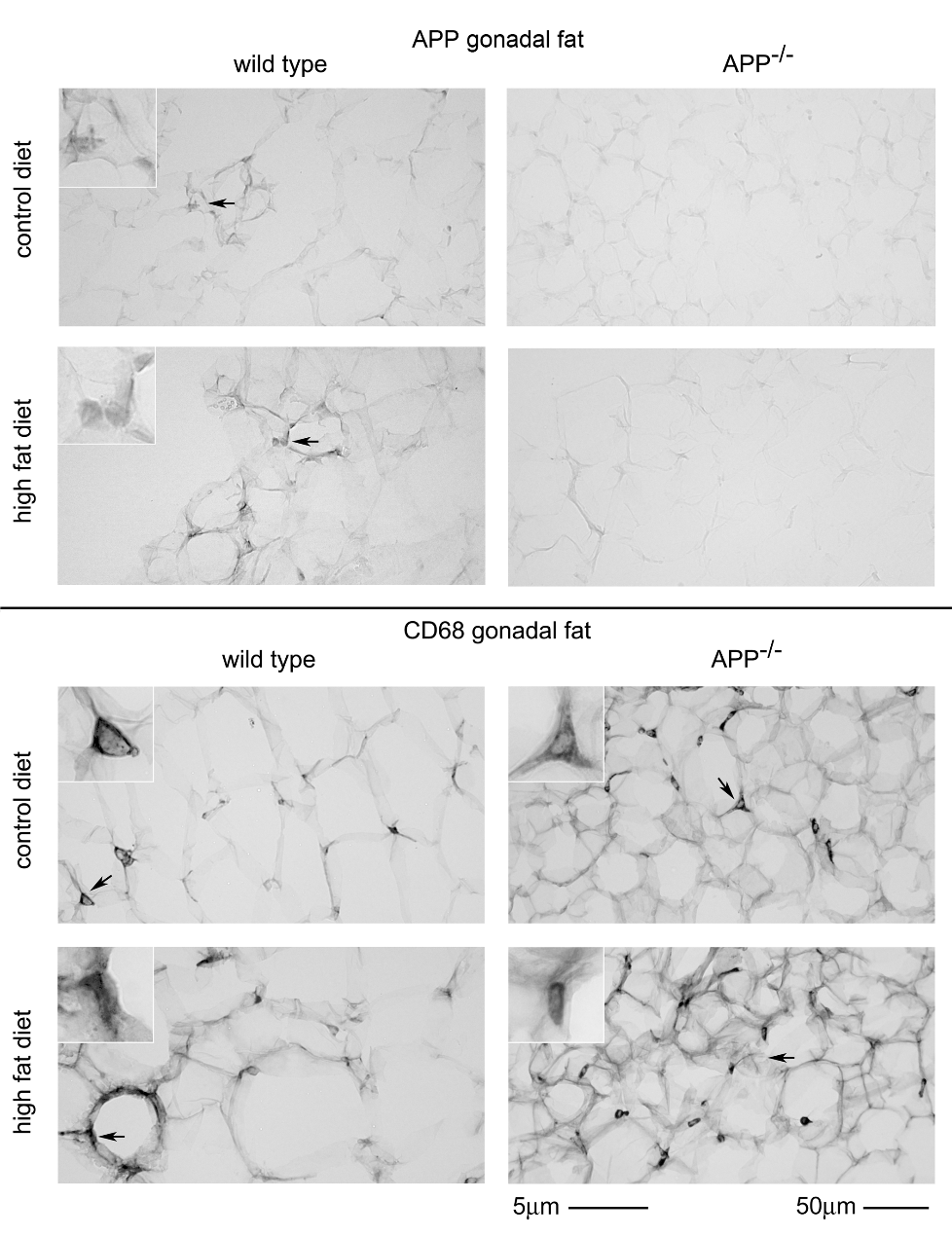
**

**
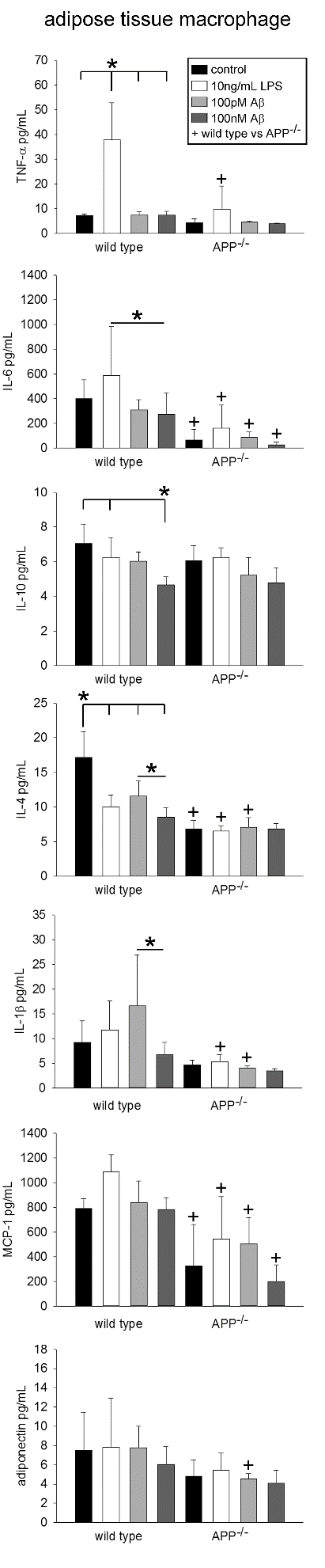
**

**Supplemental Figure 7. Adipose tissue macrophage cytokine secretion differed between wild type and APP^-/-^ cells.** Macrophages from wild type and APP^-/-^ mice were isolated from subcutaneous adipose tissue and stimulated with 10ng/mL LPS, 100nM Aβ 1-40, or 100pM Aβ 1-40 overnight and media were collected to measure IL-6, TNFα, IL-10, IL-4, IL-1β, MCP-1, and adiponectin secretion via ELISA. Data are expressed as mean +/- SD (n = 5), ^+^,*p<0.05.

**Supplemental Figure 8. Aβ levels in fat depots of control (CD) and high fat (HF) diet fed wild type mice.** Perirenal (PF), gonadal (GF), and subcutaneous (SF) adipose tissues were lysed to quantify Aβ 1-42 and 1-40 levels via ELISA. Data are expressed as mean +/- SD (n = 5 or 6), *p<0.05.


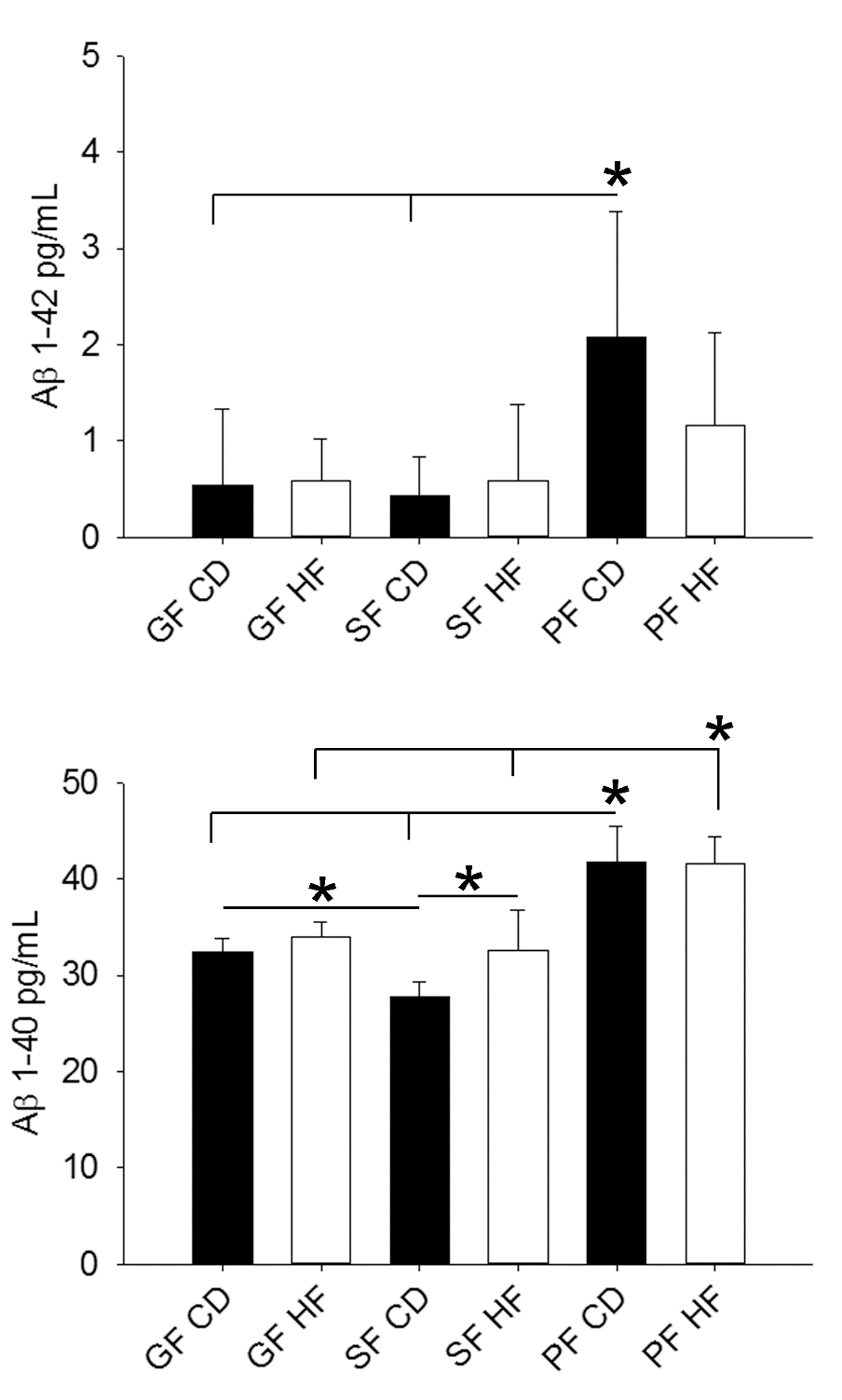


**Supplemental Figure 9. Quantification of protein levels and palmitic fatty acid uptake from wild type and APP^-/-^ adipocytes.** Subcutaneous adipocytes from wild type and APP^-/-^ mice were isolated and cultured for use. After 2 weeks in culture, adipocytes were lysed, resolved by 7-10% SDS-PAGE and western blotted using anti-APP, CD36, FABP4, FXR, LXRα, LXRβ, FAS, SREBP1, PPARγ, LRP, caveolin, adiponectin, leptin, SCD1, DLK, UCP2, GLUT-4, TLR2, TLR4, and actin (loading control) antibodies. Arrowheads indicate bands of interest when nonspecific bands are present. Antibody binding was visualized by chemiluminescence. Optical densities of the western blotted adipocyte proteins from the wild type and APP^-/-^ samples were normalized against their respective actin loading controls. Radiolabeled palmitic fatty acid (16:0) uptake was quantified from each cell type at 5, 15, and 30 minutes. Data are expressed as mean +/- SD (n = 4) ^+^p<0.05.

**
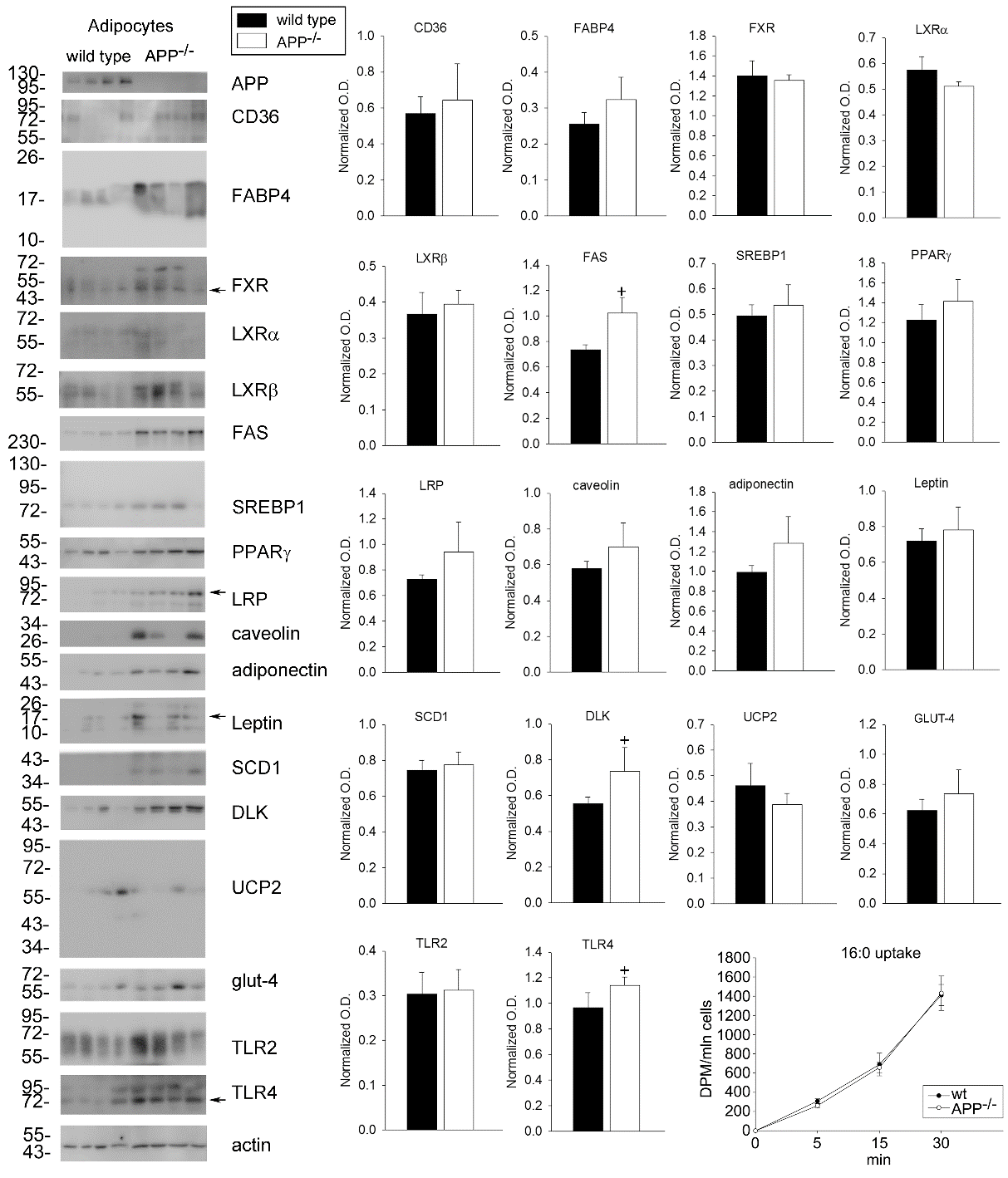
**


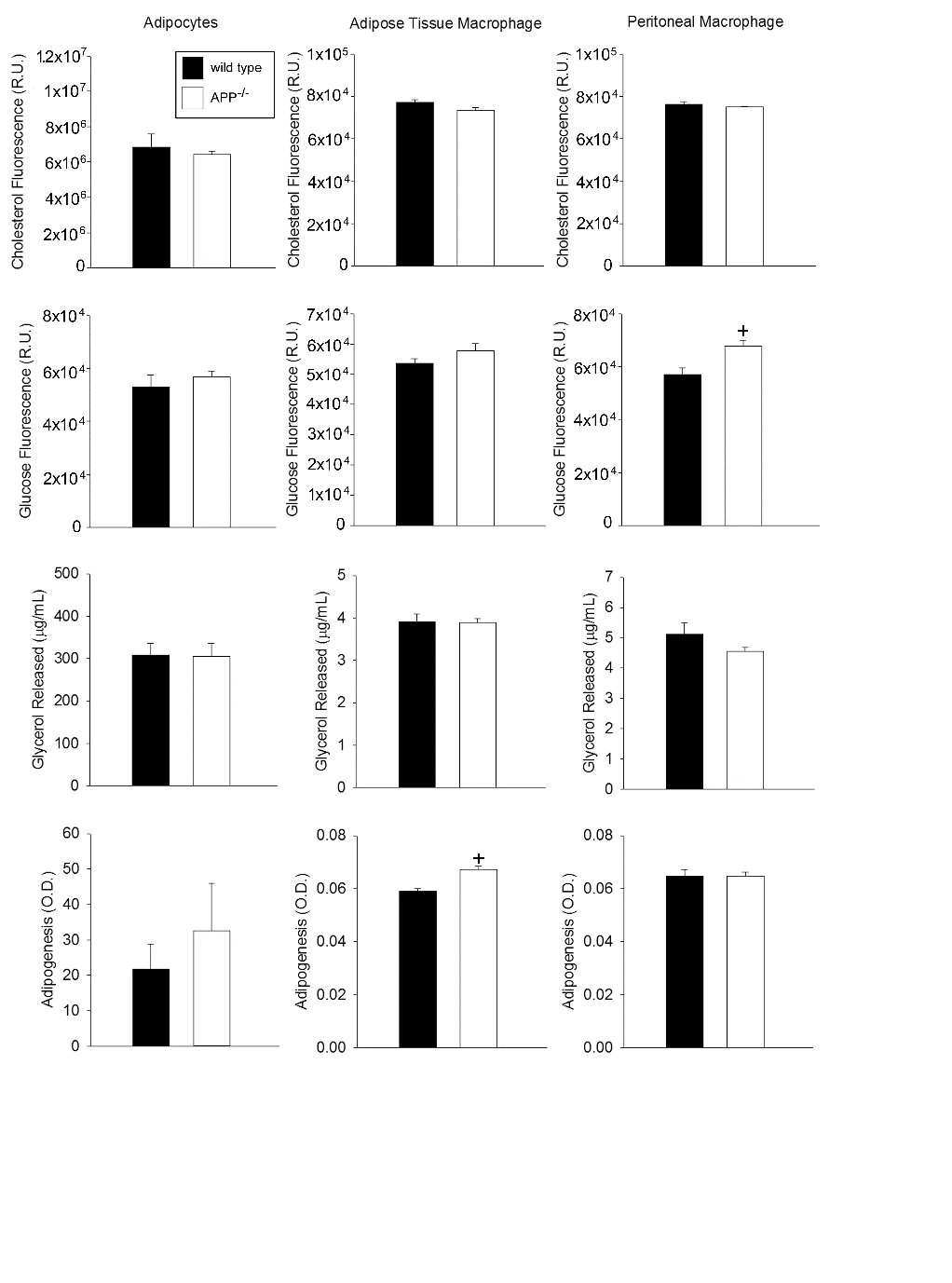
**Supplemental Figure 10. Cholesterol uptake, glucose uptake, glycerol release and adipogenesis were quantified from adipocytes, adipose tissue macrophages and peritoneal macrophages.** Subcutaneous adipocytes, subcutaneous adipose tissue macrophages and peritoneal macrophages from wild type and APP^-/-^ mice were isolated and cultured for use. Cholesterol uptake, glucose uptake, lipolysis and lipogenesis measurements were made using Cayman chemical assay kits. Data are expressed as mean +/- SD (n = 5) ^+^p<0.05.
